# The Study of Effective Factors on Iran’s Pharmaceutical Export Supply and Demand

**DOI:** 10.22037/ijpr.2019.1100841

**Published:** 2019

**Authors:** Behzad Najafi, Alireza Mahboub-Ahari, Soraya Nouraei Motlagh, Seyed Alireza Otobideh, Bahlol Rahimi, Hosein Shabaninejad, Hasan Yusefzadeh

**Affiliations:** a *Department of Health Economics, School of Management and Medical Informatics, Tabriz University of Medical Sciences, Tabriz, Iran.*; b *Department of Health Economics, School of Management and Medical Informatics, Tabriz University of Medical Sciences, Tabriz, Iran.*; c *Department of Public Health, School of Health and Nutrition, Lorestan University of Medical Sciences,Khorramabad, Iran.*; d *Department of Health Economics and Management, School of Health, Urmia University of Medical Sciences, Urmia, Iran.*; e *Department of Health Information Technology, School of Allied Medical Sciences, Urmia Medical Sciences University, Urmia, Iran.*; f *Institute of Health and Society (IHS), Newcastle University, Newcastle, United Kingdom. *; g ^*g*^ *Department of Health Economics and Management, School of Health, Urmia University of Medical Sciences, Urmia, Iran.*

**Keywords:** pharmaceutical industry, export, supply, demand

## Abstract

The importance of drug as a valuable export product in the global economy becomes clearer every day. Understanding the problems of exports and factors affecting it, can be an important step to keep Iran’s position in the world markets and further export development of this product. In this study, Iranian pharmaceutical exports’ supply and demand functions were calculated using co-integration and error correction techniques through time series quarterly data of 2000-2014 in order to identify the factors affecting pharmaceutical exports (short run and long run relationships among the variables) and price and income elasticities. The long run price elasticity of demand of -2.28 indicates that an increase in Iran’s export price relative to competitor′s export price will have a negative impact on pharmaceutical export volume. Also, the long run income elasticity of foreign demand for pharmaceutical exports of Iran is 1.11. That is an increase in income of foreign countries will have a positive impact on Iran’s pharmaceutical export. On the other hand, the long run price elasticity of export supply is 1.09, indicating that the supply of pharmaceutical export is sensitive to the relative price changes. In other words, an increase in export price relative to domestic price as well as an expansion of the pharmaceutical production capacity will increase its export supply. Being aware of the factors affecting the pharmaceutical exports can prepare the ground to develop the pharmaceutical industry and balance the supply and demand in the long run. Therefore, the results of this study can help Iran′s policymakers and managers to choose a clearer path for the pharmaceutical trade policies.

## Introduction

Promotion of non-oil exports is one of the most important measures taken to reduce Iran′s dependence on oil revenues (1). Creating new fields and increasing employment opportunities in different economic sectors, improving the quality and competitiveness of products, and utilizing the abandoned production capacities, all, require us to direct our attention toward the promotion of non-oil exports (2).

The non-oil exports leap in Iran′s Five-Year Socioeconomic Development Plan is a major goal achievable only by making use of all relative advantages of various economic sectors and making efforts and planning to retain export markets and penetrate target markets (3). 

On one hand, pharmaceuticals are the basis, and in many cases, the final point of the whole healthcare process. In other words, they complete the cycle of Iran′s Healthcare System (4). On the other hand, pharmaceuticals are considered strategic and important products in economy due to their considerable role in boosting economy and generating significant foreign exchange earnings (5). Pharmaceutical industry is classified as a hi-tech-based industry (6).

During recent years, the share of pharmaceutical products in non-oil exports has increased so that, according to the latest global reports, Iran’s rank in the Asian and global pharmaceutical markets is 4 and 12, respectively (7). 

Therefore, the promotion of science production in the field of pharmacy requires us to put the production of high-quality pharmaceutical products on our agenda. If the Iranian market is, in fact, modified in terms of management, formulation, pricing, and competition, it can significantly develop (8). 

Registration of pharmaceutical products in the destination country is an export requirement; however, Iran′s pharmaceutical exports were mainly in markets without strong, standard pharmaceutical registration (9). Failure to enter a strong pharmaceutical market is due to the absence of a registration system capable of registering pharmaceuticals based on the international laws and regulations. Accordingly, Iran has performed quite poorly in the export of pharmaceuticals (10).

The main destinations of Iran′s pharmaceutical exports are the neighboring countries, Southeast Asia and Africa. Meanwhile, Iran′s major pharmaceutical export destinations are Afghanistan and Iraq. Affordability and trust in Iranian pharmaceuticals are the two main reasons as to why the above-mentioned countries have chosen Iran as their source for pharmaceutical imports (11). 

Recently, Iran’s share in the global pharmaceutical exports has experienced a substantial decrease while, its trade competitors have seen their share grow. Therefore, due to Iran’s pharmaceutical industry’s high potentials and relative technological progress, its young, inexpensive and specialized workforce, and access to unlimited energy resource, it is quite essential to investigate the factors affecting pharmaceutical exports. This article was aimed at determining the factors affecting the pharmaceutical supply and demand. In general, export supply-and-demand functions are the bases upon which the price and income elasticities of the pharmaceutical market are determined. They are of utmost importance in designing the trade policy, determining how trade trends react to relative prices, and identifying all the other factors affecting the pharmaceutical exports (12).

*Methodology*

This research was an applied descriptive-analytical study. The statistical population consisted of economic and trade information on Iran’s business partners in the pharmaceutical sector (Afghanistan, Iraq, Ukraine, UAE, Tajikistan, Turkmenistan, Pakistan, Yemen, Uzbekistan, Armenia, Syria, Switzerland, Russia, Azerbaijan, Turkey, Sudan, Somalia, India, England, France, Germany, Jordan, and Belgium). Since few countries import pharmaceutical products from Iran, there was no need to select a sample. Census sampling was, in fact, taken into account. The data were collected from the Ministry of Industry, Mining and Trade, Iran’s Pharmaceutical Statistical Registry (Iran’s Food and Drug Administration), World Trade Organization and Trade Map website using checklists designed by the researcher (7, 13, 14).

Iranian pharmaceutical exports’ supply and demand models were analyzed using co-integration and error correction techniques in order to identify the factors affecting pharmaceutical exports (short-term and long-term relationships among the variables) and price and income elasticities. 

A- Iran′s pharmaceutical exports supply (EXPT) is supposed to depend primarily on relative export prices (REEXP) and production capacity (time). Time represents the long-term changes in production capacity. A dummy variable (D) is included to calculate the impact of export incentives and subsidies to encourage pharmaceutical manufacturers. Iran′s export activities are expected to increase due to the support provided to pharmaceutical exporters in the form of export incentives (15-19). 

EXPT_t_= ƒ (REEXP_t_, TIME, D)

The relative price of Iranian pharmaceutical exports (REEXP_t_) was calculated as a percentage of the export price index to the domestic pharmaceutical price index. In other words:

REEXP_t_= (PX_t_/PD_t_)×100

PX_t _is the Iranian pharmaceutical export Price Index, while PD_t_ is Iranian Domestic Pharmaceutical Price Index. 

The empirical equation used to estimate the Iranian pharmaceutical export supply model in the form of two logarithmic sides is as follows: 

LEXPT_t_= α_0_+α_1_LREEXP_t_+α_2_TIME+α_3_D+ε_t_

α_1_ is expected to be positive because the increase in export price compared to the domestic price encourages Iranian manufacturers to export rather than sell in the domestic market (20). α_2_ and α_3 _are also expected to be positive. For instance, the long-term production capacity expansion through technology improvement and infrastructure development and R&D along with export incentives and subsidies are responsible for the increase in the Iranian pharmaceutical exports (21).

B- The foreign demand function for Iran′s pharmaceutical exports depends on the price of Iran’s pharmaceutical export price relative to competitor’s export price and the total real GDP of the major importing countries from Iran (15, 22, 23). 

EXPT_t_= ƒ (REX_t_,GDPA_t_)

The relative price of Iran′s pharmaceutical exports (REX_t_) is calculated as a percentage of Iranian pharmaceutical export price index (PX_t_) to the average of that of competing countries (PXA_t_):

REX_t_= (PX_t_/ PXA_t_)×100

Iran’s competitors are India, China, Malaysia, Singapore, Turkey, Korea, Japan, and Hong Kong.

The double-side logarithmic form of the foreign demand function for the Iran′s pharmaceutical exports is as follows:

LEXPT_t_= β_0_+β_1_LREX_t_+β_2_LGDPA_t_+u_t_

β_1_ is expected to be negative. When the Iranian export price increases compared to that of competing countries, foreign countries would replace Iranian pharmaceutical products with those of its competing countries. β_2 _is expected to be positive because an increase in the income of the countries importing pharmaceutical products from Iran would rise the demand for the Iranian pharmaceuticals. 

For the stationarity test, generalized Dickey–Fuller test (ADF) and Johansen′s test were employed (24). In case of lack of co-integration, the unconstrained error correction modeling (UECM) was used to determine the short-term and long-run relationships between the variables included in Iran′s pharmaceutical exports models. 

## Results

The results of the Dickey–Fuller test (ADF) showed that most of the variables were not stationary. Therefore, modeling can lead to fake regression problems. As a result, the co-integration relationships among variables were investigated. Johansen′s test results showed that, in export supply-and-demand functions, some variables were co-integrated at the first degree and others were co-integrated at the second degree. Therefore, there were no co-integration relationships between the variables in export supply and demand functions. Correspondingly, unconstrained error correction model (UECM) was used to determine the short-term and long-term relationships between the variables in the pharmaceutical export model. The estimated model was tested in terms of serial correlation, functional form misspecification, normality and heterogeneity of variance. The seasonal statistics of 2000-2014 were used to estimate the functions. 

**A.** Foreign demand function for Iran′s pharmaceutical exports

Table 1 shows the results of the UECM estimation model for the Iranian pharmaceutical export model. Short-run coefficients of the differential forms of relative export prices (∆LREXT_(t-3)_) and foreign income (∆LGDPA_(t-4)_) were significant at the level of 1 and 5%, respectively.

In the long run, relative export price coefficient was significant at 10%. Foreign income coefficient had a positive significant relationship with the pharmaceutical exports at 10%. Long-term price elasticity of -2.28 showed that a 1% increase in the Iranian pharmaceutical exports compared to other competitors, assuming that other factors are constant, was responsible for 2.28% decrease in the Iranian pharmaceutical exports.

Long-term income elasticity of the foreign demand for the Iranian pharmaceutical exports was reported 1.1, meaning that 1% increase in the income of foreign countries, assuming that other factors are constant, would contribute to 1.11% increase in the Iranian pharmaceutical exports. 

**B. **Iranian Pharmaceutical Export supply Function 

Table 2 shows the results of the UECM estimation model for the Iranian pharmaceutical exports using dummy variable D (export incentives and subsidies). Although dummy variable D appeared to be positively effective in exports, it was statistically insignificant. Therefore, dummy variable D was eliminated to determine an appropriate model for the Iranian pharmaceutical export.

Table 3 shows the results of the UECM estimation model for the Iranian pharmaceutical exports in the absence of dummy variable D. In the estimated equation below, the coefficient of differential form of the relative price variable (∆LREEXP_(t-4)_) is significant at 5% level. Time is positive and significant at 1%. Long-term export price elasticity was reported 1.09, showing that the Iranian pharmaceutical exports were sensitive to the relative price. In other words, 1% increase in relative export price was responsible for 1.09 increase in the exports in the long run.

**Table 1:** Estimated UECM of Foreign demand function for Iran′s pharmaceutical exports (∆LEXPTt)

**Variables**	**Coefficients**	**Standard Deviation**	**t statistic**	**Probability**
Fixed	0.16	0.11	1.45	0.155
DLREXTt(-3)	0.208	0.069	2.98	0.005
DLGDPAt(-4)	-0.365	0.163	-2.24	0.031
LEXPTt(-1)	-0.042	0.023	-1.82	0.076
LREXt(-1)	-0.096	0.05	-1.92	0.062
LGDPAt(-1)	0.047	0.027	1.73	0.09
R2	0.8042			
Adjusted R2	0.7243			
F statistic	11.96			
Durbin-Watson statistic	2.33			
Schwartz statistic	-5.46			
Akaike statistic	-5.71			
Long run price elasticity	-2.28			
Long run income elasticity	1.11			

**Table 2:** Estimated UECM of Iranian Pharmaceutical Export supply Function with dummy variable (D).

**Variables**	**Coefficients**	**Standard Deviation**	**t statistic**	**Probability**
Fixed	-17.71	4.05	-4.37	0.0001
DLREEXPt(-4)	0.335	0.244	1.37	0.178
LEXPTt(-8)	-0.136	0.029	-4.68	0
LREEXPt(-7)	0.138	0.071	1.93	0.061
TIME	0.00002	0.000005	4.42	0.0001
D	0.0032	0.006	0.54	0.59
R2	0.7884			
Adjusted R2	0.7014			
F statistic	10.67			
Durbin-Watson statistic	2.316			
Schwartz statistic	-5.57			
Akaike statistic	-5.82			
Long run price elasticity	1.01			

**Table 3:** Estimated UECM of Iranian Pharmaceutical Export supply Function without dummy variable (D).

**Variables**	**Coefficients**	**Standard Deviation**	**t statistic**	**Probability**
Fixed	-17.71	4.94	-3.58	0.001
DLREEXPt(-4)	0.349	0.157	2.21	0.033
LEXPTt(-8)	-0.135	0.029	-4.53	0.0001
LREEXPt(-7)	0.148	0.044	3.37	0.001
TIME	0.00002	0.000006	3.54	0.001
R2	0.7857			
Adjusted R2	0.7178			
F statistic	11.24			
Durbin-Watson statistic	2.019			
Schwartz statistic	-5.66			
Akaike statistic	-5.87			
Long run price elasticity	1.01			

## Discussion

In short term, the results showed that the Iranian pharmaceutical export demand was sensitive to the changes of relative export price and foreign income. In the long run, foreign income seemed to be sensitive to the income growth of the Iranian business partners. Therefore, as the foreign income increases, demand rises for the exports. The increase in the Iranian export price was responsible for the decrease in exports. These results indicated that Iran was a small country in the world′s pharmaceutical market, not able to influence the foreign income or global prices. Therefore, future export increase needs to rely on the domestic changes, which might be gained by adopting appropriate policies in domestic industries such as R&D subsidies.

Iranian pharmaceutical export function estimation results showed that relative export price has a positive, significant relationship with the Iranian pharmaceutical export in the short run. If the relative export price rises, manufacturers are encouraged to find more attractive foreign markets and prefer pharmaceutical exports to the domestic market. As a result, the exports rise. On the other hand, if the export price decreases compared to the domestic market price, the manufacturers might find more profitable domestic markets rather than the exports. As a result, the exports decrease. 

Pharmaceutical export sensitivity to changes in relative price might be associated with the proper information flow to the Iranian exporters in terms of changes of export price compared to the domestic market price, and domestic market flexibility for export activities such as workforce and transportation. In terms of technological and infrastructural improvement, long-term production increase results in the pharmaceutical export increase.

So far, no study has been conducted on the pharmaceutical export supply and demand except for the one by Chuankamnardkan. Chuankamnardkan used co-integration and error correction techniques in order to estimate the Australian pharmaceutical export supply and demand (25). According to the results, Australian pharmaceutical export is very sensitive to the relative price (export price relative to domestic price). Export support schemes have had a positive effect on pharmaceutical exports. On the other hand, foreign demand for the Australian pharmaceutical export is very sensitive to the relative price (Australian export price to that of competitors) and foreign income, which is consistent with ours. 

Competitiveness and globalization of Iranian pharmaceutical products are low in Iran due to the Iranian low exports (11). Technological progress, product differentiation, R&D promotion, costs reduction, and economies of scale are advised to promote the Iranian pharmaceutical exports. Special emphasis on foreign direct investment (FDI), active contribution to economic convergence, and pharmaceutical industry privatization are vital recommended measures that can increase Iran’s pharmaceutical exports in the future (9, 26, 27). 

Iran′s neighboring countries such as Afghanistan, Iraq, Tajikistan, Yemen, etc. are the major importers of Iranian pharmaceuticals. It is recommended to select the prioritized pharmaceutical exporters and plan to penetrate their markets while being fully aware of the competitors’ conditions, marketing issues, and related rules and regulations in target markets. 

## Conclusion

Being aware of the factors affecting the pharmaceutical exports and following the relevant policies can prepare the ground to develop the pharmaceutical industry and balance the supply and demand in the long run. Therefore, the results of this study can help Iran′s policymakers and managers involved in the pharmaceutical industry to choose a clearer path for the pharmaceutical trade policies. 
